# HmmUFOtu: An HMM and phylogenetic placement based ultra-fast taxonomic assignment and OTU picking tool for microbiome amplicon sequencing studies

**DOI:** 10.1186/s13059-018-1450-0

**Published:** 2018-06-27

**Authors:** Qi Zheng, Casey Bartow-McKenney, Jacquelyn S. Meisel, Elizabeth A. Grice

**Affiliations:** 1 Department of Dermatology and Microbiology, Perelman School of Medicine, University of Pennsylvania, 421 Curie Blvd, BRB 1046/7, Philadelphia, PA 19104 USA; 2 Genomics and Computational Biology Program, Department of Dermatology, University of Pennsylvania, Philadelphia, USA

**Keywords:** Microbiome, 16S rRNA gene, FM-index, HMM profile alignment, Phylogenetic placement, Taxonomic assignment, Operational taxonomic unit, Dirichlet models, DNA substitution models

## Abstract

Culture-independent analysis of microbial communities frequently relies on amplification and sequencing of the prokaryotic 16S ribosomal RNA gene. Typical analysis pipelines group sequences into operational taxonomic units (OTUs) to infer taxonomic and phylogenetic relationships. Here, we present HmmUFOtu, a novel tool for processing microbiome amplicon sequencing data, which performs rapid per-read phylogenetic placement, followed by phylogenetically informed clustering into OTUs and taxonomy assignment. Compared to standard pipelines, HmmUFOtu more accurately and reliably recapitulates microbial community diversity and composition in simulated and real datasets without relying on heuristics or sacrificing speed or accuracy.

**Electronic supplementary material:**

The online version of this article (10.1186/s13059-018-1450-0) contains supplementary material, which is available to authorized users.

## Background

Culture-independent amplification, sequencing, and analysis of phylogenetic marker genes, such as the prokaryotic 16S ribosomal RNA (rRNA) gene, enables community-wide analysis of the diversity and composition of host-associated and environmental microbiota. These approaches heavily rely upon computational methods to cluster amplicon sequences into groups representing putatively conspecific sequences (operational taxonomic units [OTUs]) and to infer taxonomic and phylogenetic relationships [1]. As sequencing costs decrease and throughput increases, tools for processing and analyzing these rapidly expanding datasets must also improve with respect to speed, computational burden, and accuracy.

**Fig. 1:**
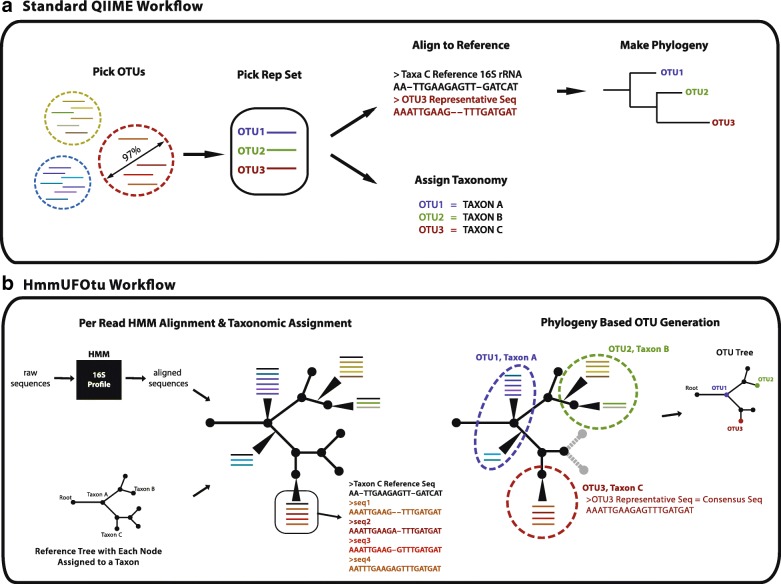
General workflow of HmmUFOtu and the default OTU-based QIIME pipeline for 16S rRNA gene sequencing studies. **a** Main steps of the default QIIME pipeline include: (1) generating OTUs (OTU picking); (2) selecting an individual read for each OTU as the representative sequence (rep-seq picking); (3) assigning taxonomic information to every OTU by comparing the rep-seq to the reference database (taxonomic assignment); and optionally (4) aligning rep-seqs to the references; (5) constructing a de novo OTU tree using aligned rep-seqs. **b** Main steps of HmmUFOtu include: (1) per-read alignment and taxonomic assignment with profile-HMM and phylogenetic placement algorithms; (2) OTU picking around existing phylogenetic nodes to generate phylogeny-based OTUs, consensus based rep-seqs, and reference-based OTU tree. *Dashed circles*: phylogeny-based OTUs; *gray dashed lines and dots*: unneeded subtrees of the reference tree that are pruned to generate the OTU tree

A typical analysis workflow first assigns sequences to OTUs, then selects a representative sequence from each OTU, and all downstream taxonomic and diversity analyses are performed with the representative sequences (Fig. 1a). Taxonomic-based analyses allow investigators to assign an identity to sequences and then infer biological and functional attributes based on relationships to previously characterized and/or cultured taxa. OTU-based analyses are agnostic to taxonomic definitions; however, current clustering methods apply similarity thresholds (e.g. 97% similarity for species-level) that erroneously assume a stable rate of evolution across the length of the gene [2, 3]. The required selection of a representative sequence from each OTU for taxonomic assignment and downstream analyses further compounds error and bias in OTU selection methods since this process precludes global optima due to the loss of information in the remaining sequences in an OTU. Together, these methods are widely used for profiling microbial communities; however, many serious practical concerns arise when considering their application to growing collections of 16S rRNA amplicon sequences.

Commonly used OTU picking methods can be classified as de novo (e.g. cd-hit, BLAST, prefix/suffix/trie, UCLUST, USEARCH, Swarm, SortMeRNA, Mothur), closed-reference (“phylotyping;” e.g. uclust_ref, usearch_ref) or open-reference (“hybrid;” e.g. [4–9]). They differ, respectively, in whether sequences are compared to each other, a fixed reference dataset, or a combination of both approaches. Because pairwise comparison of sequences becomes computationally expensive with increasing sequencing depth, present OTU picking methods often use a heuristic when comparing reads, thereby compromising accuracy and efficacy. Open-reference OTU picking is the default and developer recommended methods for OTU clustering in QIIME [10], a popular pipeline for performing microbial community analysis, though this method is well-known to result in inflated diversity estimates and to compromise the quality of the downstream data analysis [11–13].

Further compounding these sources of error and bias, presently preferred taxonomic assignment methods utilize distance-based methods, by identification of the closest reference sequence among pair-wise comparisons (e.g. BLAST, UCLUST) [6, 9] or in a multiple-alignment profile (e.g. RDP classifier) [14]. These taxonomic assignment methods have their respective advantages and disadvantages, but all neglect to incorporate established phylogenetic organization of known reference microorganisms. This exclusion can likely be attributed to the increased complexity of integrating reference phylogenetic information, due to the large number of reference sequences at a usable resolution (e.g. ~ 200,000 reference sequences in the GreenGenes 97% OTU “species level” reference tree) [15].

Here, we present a novel strategy that addresses the limitations described above in the processing of microbial amplicon-based sequencing reads for the analysis of microbial community composition and diversity. We developed HmmUFOtu, a software tool that: (1) aligns sequences; (2) places every single- or paired-end read into a known reference tree; and (3) performs phylogeny-based OTU clustering, based on either observed leaves or inferred ancestors (Fig. 1b). The revised order of taxonomic assignment followed by OTU picking circumvents many of the previously discussed limitations and enables highly accurate alignment and taxonomic assignment, selection of biologically relevant representative sequences from each OTU, and robust phylogenetic community structures, as demonstrated in both simulated and real data benchmarks.

## Results

**Fig. 2:**
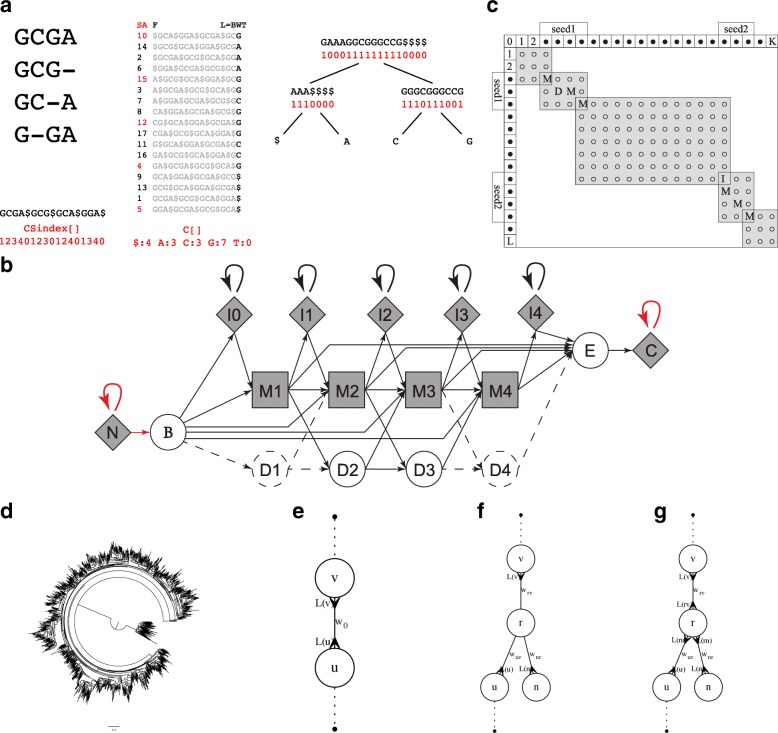
HmmUFOtu core algorithms. **a** Constructing a consensus sequence FM-index (CSFM-index) from a MSA using the Burrows-Wheeler transform (BWT) coupled with Wavelet-tree compression algorithms. *Red*: Actual stored data in a CSFM-index. **b** A “plan 7” (p7) HMM architecture specifically designed for 16S rRNA gene and other target gene/marker sequencing, with M (match), I (insertion), D (deletion), N (N′: 5′), C (C′: 3′), B (begin), and E (end) states, respectively. *Dashed circles and arrows*: “wing-retraction” process used to avoid empty alignment paths; *red arrows*: special transitions used to control the “global” or “local” alignment mode. **c** Banded-HMM Viterbi algorithm to find the most likely (minimum cost) path given the HMM profile (row), a read sequence (column), and two known “seed” paths by querying the CSFM-index. Only *shaded grids* are searched by the banded-Viterbi algorithm. The first and last shaded search areas rarely reach the profile ends. **d** An example of a 16S rRNA phylogenetic tree. In this tree, all directional conditional log-likelihoods (arrows in (e), (f), (g)) of all branches were pre-evaluated. The ancestral sequences of all internal nodes were inferred using maximum likelihood. **e** For a potential “seed” branch u--v, a small sub-tree containing only nodes u, v, the original conditional log-likelihoods L(u) and L(v) and original branch length w_0_ are copied. **f** To place a new read n to branch u--v, a new internal node r is introduced, the new conditional log-likelihoods L(n) are evaluated, then initial branch lengths w_rv_, w_ur_, and w_nr_ are estimated using observed distance (p-Dist). **g** For a candidate top estimation, the branch lengths w_rv_, w_ur_, w_nr_ and L(rv), L(ru), and L(rn) are iteratively and jointly optimized until convergence

The HmmUFOtu (Hidden Markov Model [HMM]-based Ultra-Fast OTU tool) is composed of two core algorithms: (1) a banded-HMM profile alignment algorithm (banded-HMM) utilizing a consensus sequence FM-index (CSFM-index); and (2) the Seed-Estimate-Place (SEP) local phylogenetic placement algorithm. Both algorithms are designed specifically for phylogenetic markers such as the 16S rRNA gene, in which primers designed to anneal at highly conserved regions are used to amplify a portion of a target gene containing hypervariable regions. This feature led to our design of the CSFM-index powered banded-HMM algorithm, which restrains the regular profile HMM alignment algorithm with conserved seeds at the 5′ and 3′ ends of reads (Fig. 2a–c). The SEP algorithm takes advantage of both a phylogenetic placement algorithm introduced by similar tools [16, 17] as well as our assumption that observing a novel instance of a read from a node, representing either an observed taxon or inferred ancestor, should not alter the overall topology of the known reference phylogenetic tree except for the local branch from which the read is emitted (Fig. 2d–g). Details of the core algorithms are fully elaborated in the “Methods” section.

To test HmmUFOtu performance, we built a benchmark HmmUFOtu database from the GreenGenes 97% reference database, “gg_97_otus_GTR,” using a pre-trained GTR DNA substitution model. This database roughly represents a prokaryotic (bacteria and archaea) species-level phylogenetic tree and contains ~ 200,000 nodes, of which all leaves (~ 100,000) and key internal nodes had assigned taxonomies. All unnamed internal nodes were assigned taxonomy by recursively back-tracing to their annotated ancestors.

### Simulated data

We first generated four simulated datasets from the “gg_97_otus_GTR” database. Each dataset contained sequences spanning different hypervariable regions of the 16S rRNA gene: (1) hypervariable region 4 (“V4”); (2) hypervariable regions 1 through 3 (“V1 V3”); (3) hypervariable regions 3 through 5 (“V3 V5”); and (4) a dataset containing segments drawn from random locations in the gene (“random”). We in silico “annealed” V4, V1 V3, and V3 V5 sequencing primers to the “gg_97_otus_GTR” database to obtain their consensus loci of the trained 16S HMM profile, then generated simulated reads using a procedure that mimics the construction of a real 16S library with the following steps: (1) a tree branch is randomly drawn from the reference tree; (2) a branching-point is drawn uniformly from this branch length; (3) a locus is uniformly drawn for the “random” dataset, or stays fixed at the known V4, V1 V3, or V3 V5 locus for their respective datasets (Additional file 1: Table S1); (4) amplicon size is either drawn from a truncated Gaussian distribution for the “random” dataset or fixed for V4, V1 V3, or V3 V5; (5) bases of the read are simulated according to the conditional likelihood of observing the four bases at the given branching point, or the gap probability at the consensus site (gaps are not present in the simulated reads). To replicate a practical 16S rRNA gene sequence survey, we generated 20 simulated samples, each containing 5000 in silico synthesized reads for the four datasets independently, in which both the consensus loci and the taxonomic assignment are known for all simulated reads. All four simulated datasets were then processed by HmmUFOtu and similar tools to evaluate their performance.

#### HmmUFOtu achieves both high alignment accuracy and speed

To evaluate alignment performance, all four simulated datasets were aligned to the same 16S rRNA profile using HmmUFOtu, as well as hmmalign and nhmmer, two widely used HMM aligners from the HMMER3 package [18, 19]. All three tested HMM-based tools achieved very high accuracy for the fixed locus datasets (V4, V1 V3, and V3 V5), but HmmUFOtu outperforms both hmmalign and nhmmer for the random dataset (Table 1, upper panel), suggesting our banded-HMM algorithm and HMM architecture (Fig. 2a–c) can accurately map notably divergent reads. Additionally, HmmUFOtu is also about two- to threefold faster than hmmalign and nhmmer (Table 1, lower panel), the latter of which is already an optimized algorithm for nucleotide alignment [19]. Our banded-HMM algorithm significantly improves alignment accuracy and reduces the searching space compared to existing HMM algorithms by utilizing the relatively invariant “seeds” inherent to rRNA genes.

**Table 1:** Alignment accuracy (upper panel) and speed (lower panel) of HmmUFOtu and three other aligners benchmarked with four simulated datasets

Program	Random	V4	V1 V3	V3 V5
	Alignment accuracy (%)			
hmmufotu	96.57	100.00	98.11	100.00
hmmalign	73.45	99.66	100.00	100.00
nhmmer	91.50	100.00	98.12	100.00
blastn	100.00	100.00	100.00	100.00
	Speed (reads ∙ s^− 1^ ∙ cpu^−1^)			
hmmufotu	77.1	155.1	86.8	56.2
hmmalign	24.2	38.2	26.8	21.0
nhmmer	44.6	77.5	47.6	41.4
blastn	0.228	0.228	0.222	0.220

A correct alignment is defined as the aligned consensus locus overlapping with at least 90% of the true locus. Speed is measured as reads per second per processor. All HMM aligners used their own trained HMM models from the GreenGenes 97_OTUS reference sequence alignment; NCBI blastn used the best alignment among all hits. Accuracy results are based on the aggregate of 20 replicate samples; speed results are based on the average of 20 samples

For further comparison, we also evaluated the alignment performance of a traditional pairwise alignment method using the NCBI blastn program [9]. By aligning reads to all the sequences in the gg_97_otus_GTR database and using the best alignments for every read, we found that the NCBI blastn pairwise alignment algorithm can also achieve similarly high levels of accuracy as HmmUFOtu, but at considerably slower speeds (> 300 times slower, Table 1, lower panel). This result was not surprising, since a major advantage of the HMM-profile alignment algorithm is the multiple-sequence alignment (MSA) profile size-independent processing efficiency.

#### HmmUFOtu achieves accurate taxonomic assignment for simulated reads at all taxonomy levels

We next evaluated the performance of HmmUFOtu to assign taxonomy at all levels ranging from kingdom to species using the previously described simulated datasets. Overall, we found that HmmUFOtu achieves very high assignment sensitivity (true positive rate [TPR] or recall), specificity (true negative rate [TNR]), and accuracy (ACC) at all levels (Table 2, upper panel). The assignment sensitivity drops significantly at the species level, as does the assignment precision, but still retains high levels of specificity. This result may be an artefact of potential annotation errors at the species level in the GreenGenes database; it is important to note that only ~ 1/8 of all nodes with a genus level annotation also have a species level annotation (data not shown), suggesting the difficulty in generating accurate species level annotation. At this stage, HmmUFOtu was not compared to similar phylogenetic placement tools such as pplacer or EPA [16, 17], because both tools failed to process the large reference tree due to technical errors most likely stemming from size restrictions (data not shown). Nevertheless, HmmUFOtu can perform taxonomic assignment at a speed of 3–4 reads per second per processor (Table 2, upper panel), which in conjunction with its native and effective multi-threading support, permits fast and high throughput analyses.

**Table 2:** Taxonomic assignment performance (%) and speed of HmmUFOtu and two similar tools, pplacer and EPA, at different taxonomy levels

Dataset		Performance measurement							Speed
		Kingdom	Phylum	Class	Order	Family	Genus	Species	
Random	TPR	100.00	99.96	99.52	99.22	98.87	95.03	81.96	3.14
	TNR	nan	96.98	91.76	96.04	98.09	98.09	99.35	
	PPV	100.00	99.99	99.63	99.43	99.10	95.94	84.85	
	ACC	100.00	99.94	99.19	98.82	98.62	97.10	98.62	
V4	TPR	100.00	99.96	99.52	99.26	99.17	95.78	82.75	4.61
	TNR	nan	94.85	91.17	95.93	97.98	98.17	99.44	
	PPV	100.00	99.98	99.60	99.41	99.06	96.09	86.10	
	ACC	100.00	99.94	99.15	98.84	98.79	97.40	98.77	
V1 V3	TPR	100.00	99.97	99.45	99.18	98.93	95.34	82.89	3.22
	TNR	nan	98.84	89.52	95.51	97.56	97.93	99.26	
	PPV	100.00	99.99	99.51	99.36	98.86	95.53	82.83	
	ACC	100.00	99.96	99.01	98.72	98.49	97.11	98.58	
V3 V5	TPR	100.00	99.96	99.61	99.39	99.40	96.98	86.71	3.15
	TNR	nan	98.17	94.86	97.73	99.17	98.97	99.64	
	PPV	100.00	99.99	99.77	99.68	99.61	97.78	91.05	
	ACC	100.00	99.96	99.41	99.18	99.33	98.34	99.12	
Program		Performance measurement							Speed
		Kingdom	Phylum	Class	Order	Family	Genus	Species	
HmmUFOtu	TPR	100.00	98.36	95.60	91.63	86.72	83.31	84.02	12.02
	TNR	nan	95.05	94.97	96.29	97.50	98.71	99.71	
	PPV	100.00	99.11	95.65	90.81	84.11	80.48	83.04	
	ACC	100.00	97.86	95.31	94.96	96.07	97.79	99.44	
Pplacer	TPR	100.00	99.92	99.68	99.58	99.70	100.00	100.00	3.23
	TNR	nan	69.13	74.95	80.48	87.40	93.56	98.12	
	PPV	100.00	94.77	82.16	67.11	54.72	49.75	47.74	
	ACC	100.00	95.25	88.22	85.94	89.03	93.95	98.15	
EPA	TPR	100.00	99.52	98.58	98.14	97.89	98.00	97.04	0.27
	TNR	nan	82.12	84.06	87.74	92.06	95.76	98.89	
	PPV	100.00	96.89	87.74	76.20	65.31	59.53	60.07	
	ACC	100.00	96.88	91.85	90.71	92.83	95.89	98.86	

*TPR* sensitivity, *TNR* specificity, *PPV* precision, *ACC* accuracy, *Nan* no observed data

Speed is measured as reads per second per processor. Upper panel: results from running HmmUFOtu on four simulated datasets based on gg_97_otus_GTR database; Lower panel: results from running the three tested programs on the random79 dataset based on gg_79_otus_GTR database. Speed is measured as reads per second per processor. Performance measurement results are based on the aggregate of any replicate samples; speed results are based on the average of any replicate samples

**Fig. 3:**
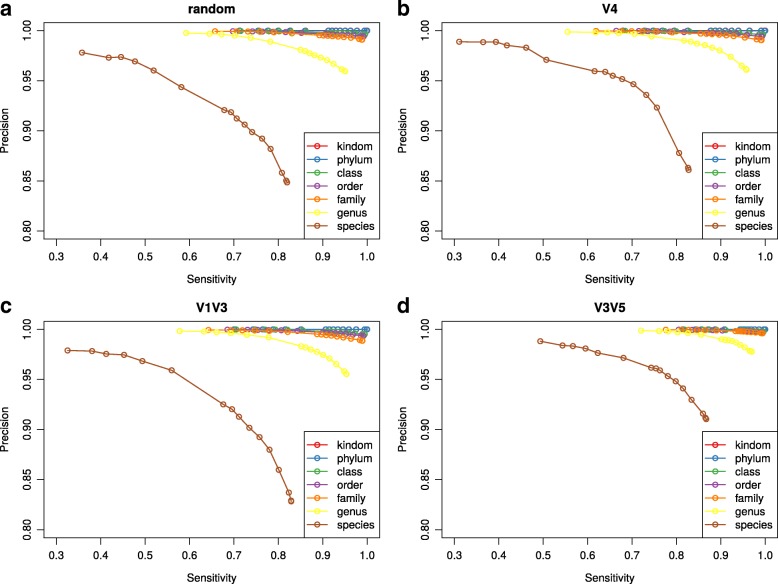
Precision-recall (PR) curves for HmmUFOtu taxonomic assignment results on four simulated datasets: (**a**) random; (**b**) V4; (**c**) V1 V3; (**d**) V3 V5. True positive (TP) and true negative (TN) are defined as both the known and assigned taxonomy having or not having a certain level of taxonomy annotation, respectively. PR curves are calculated by varying the assignment Q-score threshold from 0 to 10 with a step of 1, then 20, 30, 40, 50, 60, and 250 (the maximum value)

Although HmmUFOtu achieved high assignment accuracies at all taxonomy levels, we further investigated the balance between assignment sensitivity and precision. One concern was that incorrectly assigned taxa (low precision) may cause significant problems for downstream analyses, such as erroneous community compositions or false enrichment of “phantom” species. To this end, we generated precision and recall curves by varying the threshold of acceptable assignment Q scores calculated by HmmUFOtu. The Q-score is the posterior log-likelihood for a potential assignment to be correct given all considered assignments, which is mathematically equivalent to the “likelihood-weight” calculated by similar tools [16, 17]. As previously shown, high assignment precision was achieved at all taxonomic levels except for the species level without any Q-score filtering (Fig. 3), illustrating the inherent strength of HmmUFOtu’s algorithms. At the species level, HmmUFOtu can achieve a more satisfactory assignment precision (≥ 90%) by setting the Q-score criteria as low as 3, resulting in a modest loss of sensitivity (< 10%) (Fig. 3). Notably, the V3 V5 dataset exhibited better overall assignment performance for this simulated dataset (Fig. 3d), in agreement with previous findings where the V3 V5 region provide a more optimal combination of sequence divergence, amplicon size, and ease in primer design than the other regions tested in this study [20].

To compare the taxonomic assignment accuracy of HmmUFOtu with other phylogenetic placement tools, we built a smaller database using the GreenGenes 79% OTU data (gg_79_otus_GTR) that represents a mid-level (order/family) phylogenetic tree with only 2329 nodes, then generated a simulated dataset with 10,000 reads from random loci (random79). We did not generate similar V4, V1 V3, or V3 V5 datasets because these 16S primers were designed based on genus/species level reference sequences and thus the exact biological interpolation of such variable regions is not well defined at this higher level of taxonomy. The random79 dataset was processed by HmmUFOtu and two similar phylogenetic placement programs, pplacer and EPA [16, 17]. With the default maximum likelihood mode, HmmUFOtu exhibits 5–10% higher overall accuracy at mid-level taxonomy (Table 2, lower panel). We found that although all three tools have similarly high accuracies at the species level, both pplacer and EPA returned assignments with low precision and unexpectedly high sensitivity. This suggests that both pplacer and EPA exhibit a preference for placing sequences at lower branches of the phylogenetic tree proximal to the leaves, but the exact reason behind this behavior was not clear. In order to investigate this further, we calculated the Weighted UniFrac distance (also known as the Earth Mover’s Distance [21]) for all placements between the reference placement positions (from the simulation) and the inferred positions by HmmUFOtu, pplacer, and EPA, and found that HmmUFOtu generated placements exhibit a smaller Weighted UniFrac distance than the other two tools (0.0878, 0.0958, and 0.0921 for HmmUFOtu, pplacer and EPA, respectively). This indicates that the greater observed placement accuracies (Table 2, lower panel) were a result of pplacer and EPA placing sequences at the determined globally optimal position, which often resulted in lower taxonomic ranks. This propensity to place reads at lower taxonomic ranks can potentially result in spurious community compositions and has been actively addressed by other taxonomic assignment tools such as RDP classifier, SINTAX, and Metaxa2 [14, 22]. HmmUFOtu uses the maximum likelihood placement, or equivalently a uniform placement prior, for taxonomy assignment by default, but the users can use an alternative node-height weighted prior for preferentially assigning reads at lower (near-leaf) taxonomy levels.

In addition to improved assignment accuracy, HmmUFOtu also ran at considerably faster speeds (4- to 40-fold faster in processing speed) than pplacer and EPA on the random79 dataset, respectively (Table 2, lower panel). Notably, although the speed of pplacer and EPA cannot be strictly tested on large reference trees due to their technical limitations, the speed gain of HmmUFOtu is expected to be very profound on species resolution trees due to the linear time complexity of the two core algorithms with regard to total tree nodes [16, 17].

#### HmmUFOtu’s algorithms outperform traditional OTU-based methods for taxonomic assignment

**Fig. 4:**
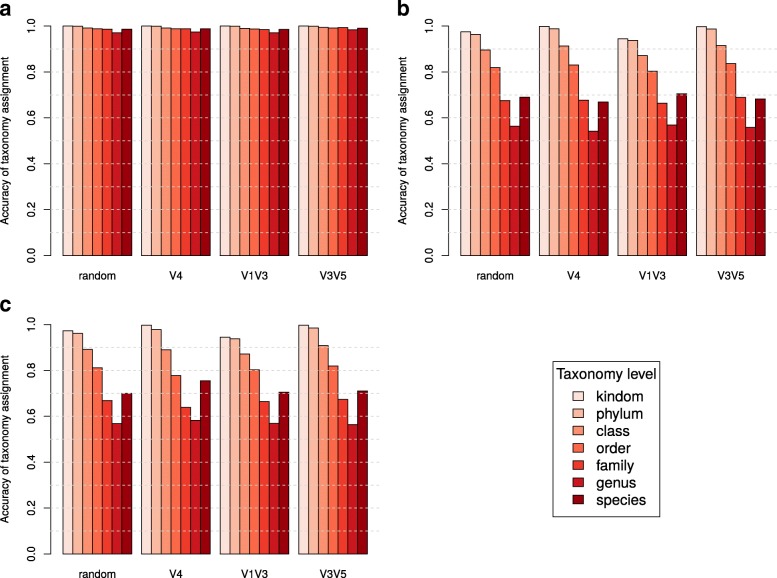
Comparison of taxonomic assignment accuracy between HmmUFOtu and uclust, the QIIME-default OTU picking strategy, using the “gg_97_otus_GTR” database. The height of the *bars* reflects the assignment accuracy of four simulated datasets at different taxonomic levels using HmmUFOtu (**a**) or QIIME-default method (**b**, **c**). **a**, **b** Accuracy measured at per-read level. **c** Accuracy measured at per-OTU level

As previously discussed, a typical analysis workflow for 16S rRNA sequence data first groups sequences into OTUs, then assigns all sequences within the same OTU the same taxonomic identity (Fig. 1). Although different in principle, both HmmUFOtu and traditional OTU-based methods eventually assign all reads to the reference taxa, giving us the opportunity to compare taxonomic assignment accuracy. We ran all four simulated datasets through the entire QIIME pipeline (v1.9.1) with default methods using UCLUST for OTU picking and taxonomy assignment and found that our profile-HMM and phylogenetic placement-based tool achieves much higher assignment accuracy at all phylogenetic levels (Fig. 4a, b). In contrast, the QIIME-default method only achieves similarly high accuracy at the higher taxonomic levels (phylum to order), suggesting the potential for a significant proportion of sequences being assigned to incorrect genus or species by traditional OTU-based methods.

In order to determine which step(s) of the OTU-based methods, namely OTU-picking and taxonomic assignment, might cause such assignment errors, we evaluated the assignment accuracy of QIIME-default methods at the OTU level, for which one representative sequence from each OTU was analyzed. At the OTU level, the assignment accuracy only slightly increases over assignment at the per-read level (Fig. 4c), suggesting the traditional distance-based taxonomic assignment methods as the primary contributors of assignment error compared to a phylogenetic placement-based method. We also observed a modest increase in assignment accuracy at the OTU level for the V4 dataset (Fig. 4c vs. b), which contains the least sequence diversity. This highlights the impact of the OTU-picking step on overall assignment accuracy, especially for less divergent datasets where reads are often grouped into larger OTUs.

### Application on real data

Real microbiome datasets are more complicated than simulated datasets, as they can be paired-ended, are subject to polymerase chain reaction (PCR) and sequencing errors, contain large amplicons with non-overlapping mates, or contain other experimental errors. Additionally, the true taxonomic identities of real datasets are usually unknown, making it impossible to adequately analyze the accuracy of read-level taxonomic assignment. In this study, we further benchmarked HmmUFOtu and similar tools using real datasets consisting of: (1) 16S rRNA amplicon datasets generated from a synthetic “mock” bacterial community DNA sample (“mock dataset;” Additional file 1: Table S2); and (2) publicly available 16S rRNA amplicon datasets generated by the Human Microbiome Project (“HMP dataset;” Additional file 1: Table S3).

#### HmmUFOtu closely recapitulates mock community composition and diversity

DNA isolated from a synthetic mock bacterial community was amplified and sequenced using either the V4 or V1 V3 protocol as part of every Illumina MiSeq run performed by our group. The mock community contains genomic DNA of 20 different bacterial species belonging to 17 different genera with equivalent concentrations of 16S rRNA genes from each species (100,000 copies per organism per μL). We used the pre-processed V4 and V1 V3 mock community datasets with forward/reverse reads assembled by Pear v0.9.0 [23] for analysis with both HmmUFOtu and the QIIME-default method. The relative community compositions at the genus level were calculated for each analysis with the generated OTU tables. Due to a built-in feature for processing unmerged paired-end reads (see “Methods”), we also processed the raw unassembled paired-end V4 and V1 V3 reads with HmmUFOtu.

**Fig. 5:**
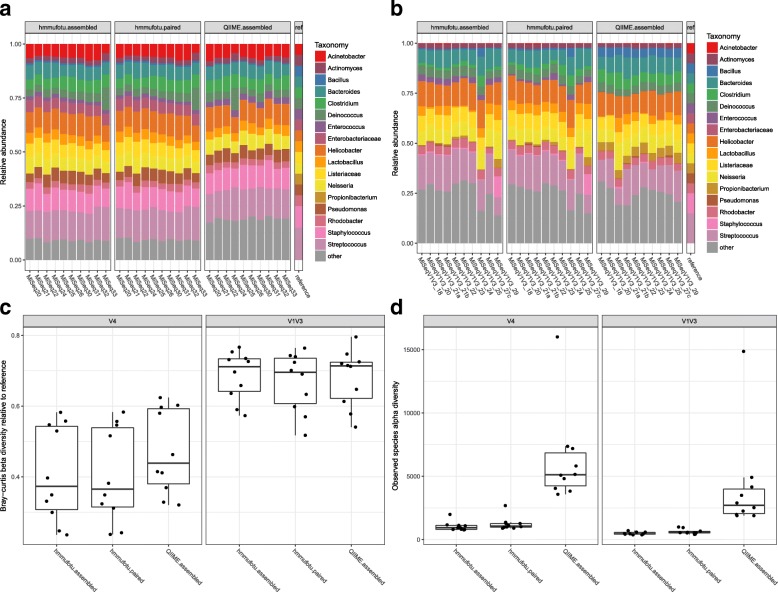
Comparison of inferred bacterial community structures between HmmUFOtu and QIIME-default methods using V4 and V1 V3 mock community datasets. Both mock datasets contain ten replicates sequenced across ten different Illumina MiSeq runs. **a**, **b** Inferred and theoretical () mock community compositions for V4 and V1 V3 datasets, respectively, calculated using HmmUFOtu or QIIME-default generated OTU tables. *Bars*: replicate samples; assembled: pre-processed paired-end merged reads; paired: un-processed paired-end reads. **c** Community structure dissimilarity between the inferred and reference community structure as calculated by the Bray-Curtis beta-diversity metric. The median is represented by the *line* in the *box*, *hinges* represent the first and third quartiles, *whiskers* represent 1.5 times the interquartile range, and *dots* represent outlying data points. **d** Alpha-diversity of the mock community measured by the inferred number of observed species. *Box plots* are as above. *Left panels*: V4 datasets; *right panels*: V1 V3 datasets

When comparing the OTU tables generated by each analysis to the theoretical composition of genus-level mock community taxa, we found that HmmUFOtu closely recapitulated the compositions of the reference community, especially for the V4 mock dataset (Fig. 5a, b). This similarity remains stable across the replicate samples that were sequenced on different Illumina runs, as revealed by the relatively invariant beta-diversity dissimilarity between HmmUFOtu inferred and reference compositions (Fig. 5c), indicating that our algorithm is robust to experimental conditions.

We also found that while both HmmUFOtu and QIIME-default methods revealed similar mock community compositions, HmmUFOtu assigns fewer reads to taxonomies outside of the 17 reference genera, especially for the V4 dataset (Fig. 5a). We further scrutinized these results by comparing alpha diversity as measured by the number of observed species, independent of the known taxonomic assignment of the reference taxa (Fig. 5d). The QIIME-default method consistently inflated the number of observed species compared to HmmUFOtu (Fig. 5d, Kruskal–Wallis test; *p* < 3.7e-5 and *p* < 3.8e-5 for the V4 and V1 V3 dataset, respectively). HmmUFOtu also exhibited stability between different sequencing and processing strategies (assembled vs paired, V4 vs V1 V3, Fig. 5d). Additionally, we found that using unassembled paired-end reads with HmmUFOtu produced slightly more accurate and stable community structures (Fig. 5c, d) compared to assembled reads. Given that up to 5% of all reads could be lost during the paired-end merging step (data not shown), we strongly recommend the input of quality trimmed unassembled, demultiplexed paired-end reads.

#### HmmUFOtu’s phylogeny-based OTUs and consensus-based representative sequences better capture the real bacterial genomic sequences

**Fig. 6:**
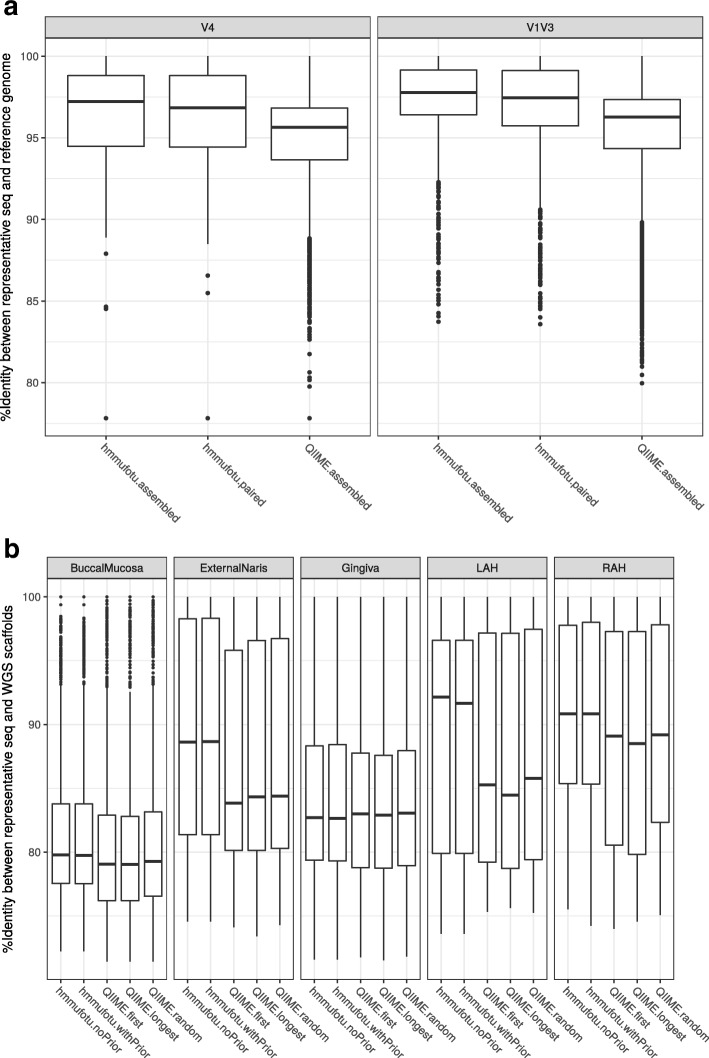
Comparing the quality of rep-seqs between HmmUFOtu’s consensus-based and QIIME’s single-sequence based rep-seq picking methods. For HmmUFOtu: the consensus sequences with (default) or without the priors were tested; for QIIME: the “first,” “longest,” and “random” methods were tested. **a** Mock datasets, in which the quality is reflected by the %identity between the rep-seqs and the known bacterial reference genomes. **b** HMP datasets, in which the quality is reflected by the %identity between the rep-seqs and the de novo assembled scaffolds from the WGS data sequenced in the same samples. LAH left auriculotemporal part of head, RAH right auriculotemporal part of head

HmmUFOtu generates phylogeny-based OTUs using the basic assumption that each tree node would become its own OTU, as they are either true observed (leaf) or inferred ancestor (internal) sequences. Therefore, any phylogenetic tree node with reads placed closest to it becomes a phylogeny-based OTU (Fig. 1a). Instead of picking one sequence as the representative sequence of an OTU, we take advantage of both pre-aligned reads (by the banded-HMM algorithm) and pre-evaluated Log-Likelihoods (by the SEP algorithm) of the OTU, and then use a Bayesian statistical model to infer the consensus sequence of the observed OTU node (see “Methods”). By aligning the generated representative sequences (rep-seqs) to the known genomic sequences of the mock community bacteria (Additional file 1: Table S2) using NCBI blastn program [9], we found that the HmmUFOtu’s consensus based rep-seqs show a higher sequence similarity (percent identity) to the reference genomes compared to the QIIME-default method that uses the first read (“first”) in each OTU (Fig. 6a, Kruskal–Wallis test; *p* → 0 for both V4 and V1 V3 datasets), suggesting the consensus of all observed sequences generally better represents the true bacterial target gene sequences by aggregating information across multiple reads and/or samples.

To further confirm that the known reference genome sequences correctly reflect the real 16S rRNA gene sequences and do not suffer from incorrect annotations or other errors, we compiled a second real dataset from the Human Microbiome Project (HMP) which contains ten samples from five distinct body sites interrogated at two independent visits using the Roche/454 Next-Gen Sequencing (NGS) platform (Additional file 1: Table S3). These samples were selected because they all have matched whole genomic shotgun (WGS) sequencing data available that were sequenced using the same platform (Additional file 1: Table S3). Similar to the mock community dataset results, we found that HmmUFOtu generates rep-seqs that more closely resemble the assembled microbiota scaffolds from the WGS data compared to the QIIME-default “first” OTU-picking method (Fig. 6b). In addition, two other rep-seq picking methods from QIIME, “longest” and “random,” similarly produce rep-seqs with lower sequence identity to the WGS-based scaffolds. Also, we failed to detect a significant difference in accuracy when HmmUFOtu either utilized or ignored prior phylogenetic information to infer the consensus-based rep-seqs. Overall, we found that using the consensus sequences across multiple reads produces more accurate rep-seqs compared to single-read based methods (Fig. 6b, Kruskal–Wallis test; *p* < 7.6e-10). Moreover, HmmUFOtu can construct the phylogenetic tree of OTUs (OTU-tree) in negligible time compared to the de novo tree construction method used by the QIIME pipeline (data not shown), which is achieved by simply pruning the reference tree to exclude subtrees without any placed reads (Fig. 1b).

### Detection of chimeric sequences

**Fig. 7:**
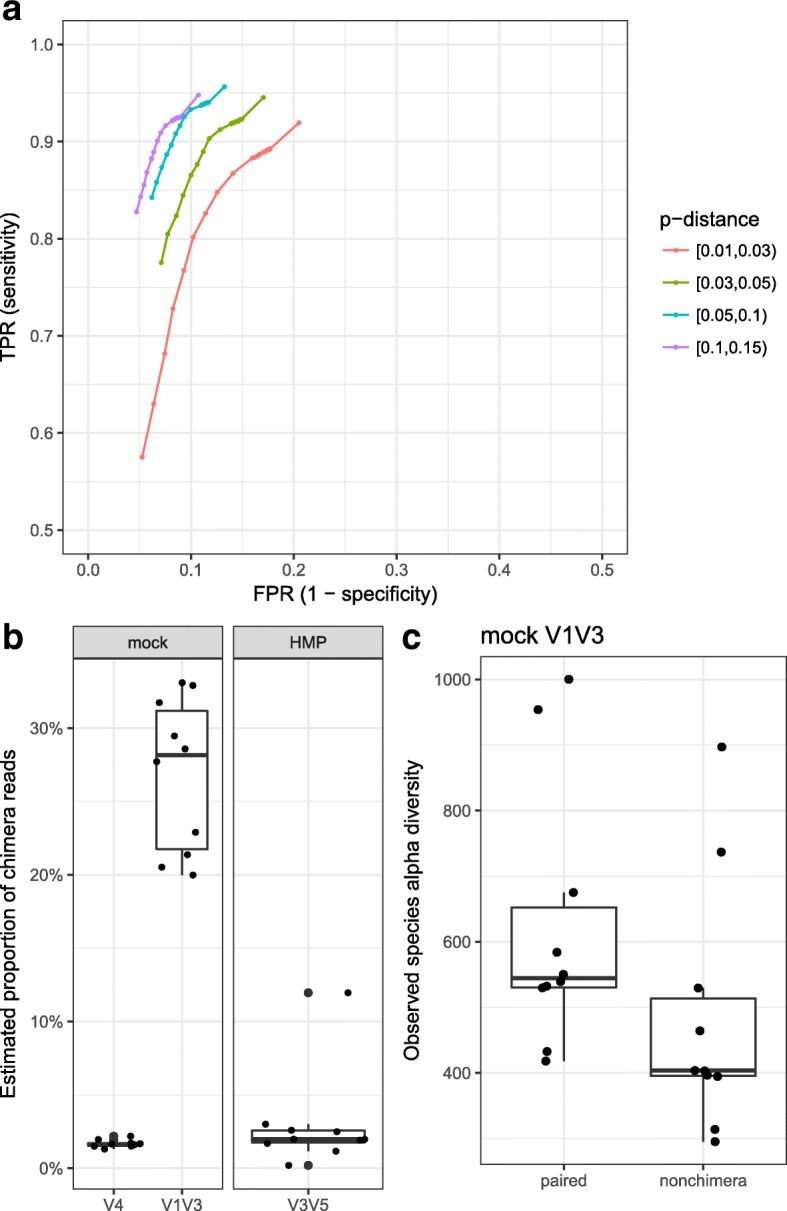
Benchmarking results from chimeric read detection using the “segment placement comparison” algorithm from HmmUFOtu. **a** Receiver operating characteristic (ROC) curves for detecting simulated chimeric reads from random in silico cross-over events using GreenGenes 97% OTU reference sequences; 10,000 chimeric or non-chimeric simulated reads were used in the respective range for each p-distance subset. ROC curves are calculated by varying the min LOD cut-off from 0 to 10 with a step of 1, then 20 to 100 with a step of 10. **b** Estimated proportion of chimeric reads in all of the benchmarked real datasets (mock and HMP) by enabling HmmUFOtu’s chimera detection and setting the LOD cut-off at 50. **c** Differences in “observed species” alpha diversity of the mock community V1 V3 dataset. Paired: original results using raw paired reads; Nonchimera: chimera-filtered results using the same paired reads

Amplicon sequencing of highly conserved marker genes such as the 16S rRNA gene can produce “chimeric” DNA sequences, which are often caused by an unintentional ligation of two distinct but closely related DNA templates during the PCR amplification step [24]. The summarization of the placement log-likelihood of our SEP algorithm over all aligned regions may be biased by chimeric reads, resulting in incorrect phylogenetic placement at some common ancestor of the two initial templates, subsequently introducing artificial and erroneous OTUs. In order to detect potential chimeric reads, we developed a “segment placement comparison” algorithm that borrows the idea of “segment alignment,” a method commonly used in other published chimera detection tools such as ChimeraSlayer and UCHIME [24, 25] (see “Methods”). Briefly, HmmUFOtu compares the joint log-odds (LOD) of alternative vs best placement of both a 5′ and 3′ segments. For benchmarking performance, we generated a simulated dataset of chimeric sequences from the GreenGenes 97% OTU reference sequences (“gg_97_otus_chimera”) using a procedure similar to the method described by UCHIME [25] involving the following steps: (1) a pair of reference sequences are randomly selected from the GreenGenes 97% OTU database, as long as the p-distance between their aligned sequences falls into any of the four ranges (i.e. [0.01,0.03), [0.03,0.05), [0.05,0.10), [0.10,0.15)); (2) a breaking point is uniformly drawn at their aligned (consensus) positions; (3) if the breaking point is within [0.25,0.75), the two reference sequences are in silico spliced and ligated into a chimeric sequence, otherwise the first sequence is kept unchanged; lastly, gaps from the alignment of chimeric and unchanged sequences are removed, whereby the resulting sequences are used as the simulated dataset. When testing performance, we found HmmUFOtu’s “segment placement comparison” algorithm could reliably identify chimera sequences with ~ 90% accuracy even with a minimum LOD threshold of zero (0) (Additional file 1: Table S4). As expected, the overall accuracy of chimera detection drops as the similarity between sequences increases (smaller p-distance); the accuracy dropped to 85% in the most extreme case (p-distance in [0.01,0.03), Additional file 1: Table S4). The sensitivity is consistently > 90% at this cut-off, comparable to previous reported sensitivities (> 70%) on different simulated datasets [24, 25], which supports the efficacy of our segment placement-based algorithm. We then compared the chimera detection sensitivity and specificity curves (ROC curves) of our algorithm by varying the minimum LOD cut-offs to ensure minimal classification of non-specific (false-positive) chimeric sequences, which can cause a significant loss of information. As shown in Fig. 7a, the specificity can be improved to > 90% (FPR or false positive rate < 0.1) by setting a minimum LOD cut-off as low as 50 with only a modest sensitivity loss (< 10%). This trend was also observed in the [0.01,0.03] sub-dataset that are most difficult to detect given the similarity in the two initial sequences (Fig. 7a, red curve). Thus, we set the default LOD cut-off for chimera detection at 50 for specific results with high retention.

In order to estimate the proportion of chimeric results in the real datasets we previously analyzed, we re-processed all the mock and HMP datasets by enabling the chimera-detecting algorithm using the recommended LOD cut-off (50). Surprisingly, the mock V1 V3 dataset exhibits a consistently high estimated proportion of chimeric reads in the range of 20–30%, which is much higher than either the mock V4 dataset (2–3%) or the HMP dataset (5–10%) (Fig. 7b). Although there is no clear explanation for the discrepancy in proportions of chimeras, we speculate that higher rates stem from deeper sequencing depths (data not shown), larger consensus amplicons sizes, and the less efficient annealing properties of the forward primers used in the mock V1 V3 dataset (Additional file 1: Table S1).

We have suggested that chimeric reads likely lead to artifact OTUs during analysis. To verify this, we regenerated the phylogeny-based OTUs with the chimera-filtered results and found the overall alpha-diversity of the communities, as measured by the “observed species” metric, decreased proportionally with the number of chimeric reads (Fig. 7c), suggesting that most chimeras produced singleton OTUs in the original unfiltered analysis.

### Multi-threading performance and vectorization

One technical limitation of traditional OTU-picking methods is the difficulty in parallelizing jobs due to the pairwise-comparisons inherent to most methods. Furthermore, parallelization of the taxonomic assignment step is often implemented externally by utilizing UNIX sub-processes that dramatically increase the hardware burden. In contrast, the HmmUFOtu core algorithms are designed to be independent for each read (see “Methods”) and can thus be easily parallelized (multi-threaded) across multiple reads. The native multi-threading support also reduces the requirement of hardware resources, which is critical for very large inputs.

**Fig. 8:**
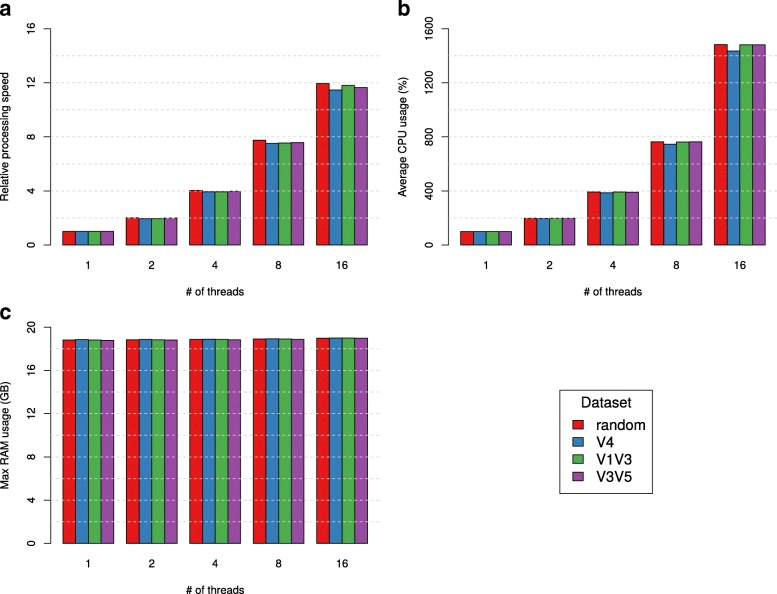
Multi-threading performance of HmmUFOtu benchmarked on four simulated datasets on 1–16 threads. **a** Relative processing speed (reads per second) normalized to 1-thread results. **b** Average CPU usage (%). **c** Maximum RAM (memory) usage in GB. All results are based on the average of 20 replicate samples

We benchmarked the multi-threading performance of HmmUFOtu using one simulated sample containing 5000 reads from each of the four simulated datasets with 1, 2, 4, 8, or 16 threads. We found that the processing speed of HmmUFOtu increased almost linearly to the number of threads up to the delegation of eight threads (Fig. 8a). When employing 16 threads, the linear relationship was most likely lost due to the restriction of total available physical cores (12 in total) on the benchmarking machine. This is illustrated by the near-linear increase of the average CPU usage (Fig. 8b). Notably, HmmUFOtu only requires a near-constant RAM regardless of the CPU usage or the type of data (Fig. 8c), thus in theory, permitting the program to handle much larger 16S datasets generated in the future without requiring an equivalent demand in RAM.

Vectorization is a programming technique based on processor supplementary instruction sets available on many processors (such as Intel and AMD), which can significantly improve the processing speed of mathematical operations especially for vector and matrix operations. HmmUFOtu utilizes the built-in vectorization ability of Eigen3, which supports SSE2 and higher standards. For example, HmmUFOtu can quickly perform the matrix exponential calculations required by the GTR DNA substitution model, comparable in time to the processing of less complex DNA models that are based on simple tractable numerical calculations (TN93 and HKY85, Additional file 1: Table S5).

### Implementation of multiple DNA and mutation rate models

The previously described analyses, including those performed on both simulated and real datasets, were processed using the gg_97_otus_GTR database, which uses the default generalized time-reversible (GTR) DNA substitution model to evaluate the likelihood of the reference phylogenetic trees [26, 27]. Although the GTR model is used by the GreenGenes database [15], we further tested HmmUFOtu on other popular models, since these simpler DNA models, such as TN93 [28] or HKY85 [29], are generally more robust due to the smaller number of free parameters and mathematically tractable features. We compared the GTR, TN93, and HKY85 DNA models using the V4 simulated dataset with the same methods described above and found that all models exhibit very similar assignment performance regarding the sensitivity, specificity, precision, and accuracy at all taxonomy levels (Additional file 1: Table S5). Notably, the simpler models TN93 and HKY85 often lead to slightly better assignment results even if the reference tree was built using the GTR model (Additional file 1: Table S5), suggesting that by using fewer free parameters, these models can still capture sufficient information from the phylogenetic structure of bacteria.

In addition to the different DNA models, we also studied the heterogeneity of mutation rates among different 16S rRNA gene sites by capturing the rate variation among sites using Discrete Gamma Distribution (*dΓ)* based models [30] (see “Methods”). Interestingly, by allowing variation among sites using the *dΓ* model, the assignment sensitivity and precision both experience significant loss compared to the fixed rate models (Additional file 1: Table S5), potentially due to the construction of reference trees in GreenGenes, which assumed fixed rate models [15]. Considering that the processing speed is much slower (~ 1.5× slower at default settings; Additional file 1: Table S5), we strongly recommend against using a Gamma model of among site rate variation for reference trees built with fixed rate models. Further, the estimated shape parameters of the *dΓ* models are usually around 0.5, indicating that most 16S rRNA gene sites are almost completely “invariant” (constant regions), while “mutation hotspots” exist in limited sites (hypervariable regions) [31], recapitulating previous estimations of the shape parameters in a study using multiple methods [32] (Additional file 1: Table S5).

## Discussion

Traditional workflows based on distance-based approaches first group sequences into OTUs, then assign taxonomy to (often arbitrarily) selected representative sequences. These methods are widely adapted due to the straightforward nature of their concepts and relatively well-established algorithms; however, they suffer from major drawbacks such as non-optimized assignments, sensitivity to input data order, limited statistical underpinnings, and disregard of the phylogenetic nature of bacterial sequences [1, 8, 12, 33]. Many of these shortcomings stem from circumventing computationally expensive algorithms and complex implementations of related methods in favor of faster heuristics.

We highlight the shortcomings of these methods by comparing HmmUFOtu to the default methods implemented by the widely used QIIME platform [10]. In both real and simulated datasets, HmmUFOtu outperforms QIIME default methods in accurately recapitulating microbial community composition and diversity. Notably, HmmUFOtu produces results with biologically realistic metrics regarding diversity and selects rep-seqs that more accurately represent the consensus sequence of the OTU. We also heavily employ prior knowledge in our tool, such as searching for “seed” alignments in the banded-HMM algorithm, as well as the stepwise SEP algorithm that uses the inherent phylogenetic nature of 16S rRNA genes.

HmmUFOtu organizes amplicon reads into OTUs by grouping together reads that have been placed around reference phylogenetic nodes. This procedure is independent of sample size and input order, two features that can affect traditional OTU-based methods, resulting in error-prone and slow analyses [1]. Additionally, because each sequence is individually processed by HmmUFOtu, subsets of samples can be pre-processed before the collection of all samples; users would only need to summarize over all pre-processed assignment files to initiate downstream analyses, a step that generally takes only minutes. However, one limitation of HmmUFOtu to consider is the operation within constraints of a reference tree. References will be biased toward those organisms that are readily culturable, may not fully represent the biodiversity of a community, and may lead to generic taxonomic assignment at very high levels (phylum or class) [11].

The algorithms implemented in HmmUFOtu are both powerful and flexible enough to be extended into microbiome research beyond the prokaryotic domain. Similar conserved marker genes exist in other systems, such as the eukaryotic 18S rRNA gene and fungal ITS region, from which amplicon sequencing data are rapidly accumulating. However, the current standard databases for eukaryotic marker genes are not as well annotated as the 16S rRNA gene databases; for example, the “SILVA” database for the eukaryotic 18S rRNA gene only provides a “guide-tree” for their sequences, which lacks branch length information and is not bi-sectional [34, 35]. Further, the “UNITE” database for the fungal ITS region does not provide any phylogenetic tree due to the high variance of ITS sequences [36]. Until fully evaluated and expert-curated phylogenetic trees from these databases are released, traditional OTU-based tools such as QIIME [10] and Mothur [5] will still be the preferred choices for analyzing eukaryotic amplicon sequencing data. A potential application for future available eukaryotic marker gene databases (i.e. ribosomal small subunit database) would be the ability to distinguish between prokaryotic and eukaryotic sequences in a sample as to remove the “contaminating” mitochondria and chloroplast 16S (m16S and c16S) sequences often found in environmental and fecal samples. However, presently, users are encouraged to use other non-phylogenetic based tools such as Metaxa2 [22] for pre-filtering eukaryotic m16S and c16S sequences.

While researchers increasingly employ metagenomic and other multi-omic approaches for inferring characteristics of microbial communities and their interactions with the host and environment, amplicon sequencing of the 16S rRNA gene remains a staple in the toolkit of microbial community ecology. HmmUFOtu considerably improves upon current methods that form the core of popular analysis workflows by incorporating phylogenetic information into the crucial OTU-picking step and thereby improves the quality of downstream analyses and interpretations.

## Conclusions

Here we describe a novel method for resolving a fundamental problem in 16S rRNA gene amplicon and other target-gene based microbiome research – clustering sequences into biologically relevant OTUs and correctly assigning taxonomy to all reads within the OTU. We achieve this using two core algorithms: a CSFM-index powered banded-HMM algorithm and a SEP local phylogenetic placement algorithm. Our banded-HMM algorithm achieves high accuracy when aligning to a 16S rRNA gene sequence MSA, similar to other HMM-based aligners, but does so at 2–3 times the speed. The SEP algorithm achieves high accuracy, sensitivity, specificity, and precision in taxonomic assignment performance, even at species-level resolution for both simulated and real datasets, at speeds up to 30 times faster than current phylogenetic placement tools [16, 17]. Taken together, HmmUFOtu can perform HMM-profile alignment, phylogenetic placement-based taxonomic assignment, phylogeny-based OTU picking, and consensus-based representative sequence inference in a species-resolution reference tree with ~ 200,000 nodes for 1 million 16S rRNA gene amplicon sequences within 6 h on a modest Linux workstation or cluster with 16 processors, 32 GB RAM, and ~ 20 GB disk requirement.

## Methods

### CSFM-index powered banded-HMM alignment algorithm

The banded-HMM alignment algorithm is designed to align NGS reads to a MSA profile with the assistance of short anchored segments, or “seeds,” along a known alignment path. To achieve this, the algorithm first attempts to find up to two seeds at the 5′ and 3′ end of a read using a CSFM-index data structure (Fig. 1a). The CSFM-index is a modified version of an FM-index (Full-text index in Minute space, a compressed full-text index based on the Burrows-Wheeler Transform) [37] that stores additional information for quickly locating the consensus sequence (CS) position in an MSA profile. Our CSFM-index implementation also utilizes a Wavelet-tree for indexing BWT strings [38], thus resulting in a small memory footprint and fast search speeds.

In order to accurately align NGS reads to the MSA profile, HmmUFOtu employs a widely used HMM architecture with seven special states (“plan7”) to capture the site-specific transition and emission features (Fig. 1b), which mimics the same core model used by the HMMER3 package [18]. However, our “plan7” architecture includes modifications designed for 16S rRNA gene sequence alignment, specifically, limiting alignment of reads to one 16S profile, rather than aligning against multiple profiles, via the “J” state. The model has also been modified for unbiased local-alignment regarding the HMM profile by allowing direct entrance into intermediate match (“M”) states with comparable probabilities. Additionally, our architecture introduces the I_0_ and I_K_ states representing the frequently observed 5′ and 3′ overhangs in 16S rRNA sequence reads stemming from non-conserved 5′ and 3′ tail regions in some bacteria species.

Once the 5′ and 3′ seed alignment paths are identified by CSFM-index searches, HmmUFOtu uses a banded version of the Viterbi algorithm to find the global optimal alignment between the NGS read and HMM profile in or near these seed regions (Fig. 1c, gray regions), which maximizes the log-likelihood (equivalently, minimizes the cost) of observing the read sequence given the trained HMM-profile (Fig. 1c) using Dynamic Programming (DP). The banded HMM algorithm Procedure-Banded-HMM is further explicated below.

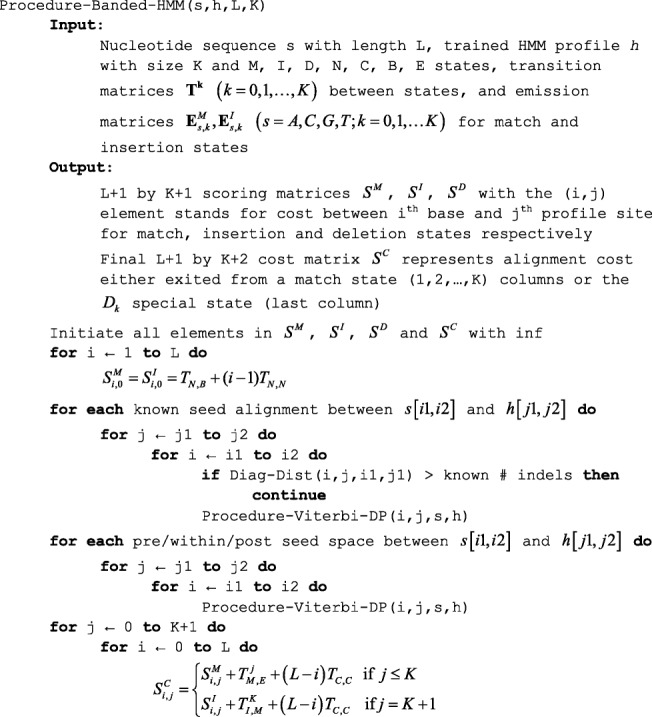



where Diag-Dist and Procedure-Viterbi-DP are described in the Appendix.

It is of note that for a very small proportion of NGS reads, no seed-alignment can be found by CSFM-index searches; we handle these reads with the standard, non-banded, HMM algorithm, as described previously [18]. For paired-end reads, the forward and reverse mates were independently aligned using our method, then the alignments are merged as if a single assembled read were used, regardless of whether the forward or reverse mates have overlapping tails. To assess the alignment accuracy, a correct alignment is defined as overlapping ≥ 90% of its true locus, a commonly used threshold in many similar situations as described previously [39, 40].

### SEP local phylogenetic placement algorithm

Once a NGS read is aligned to the MSA profile using the banded HMM algorithm, HmmUFOtu uses a local phylogenetic placement-based method to assign the correct taxonomic identification to a read based on the alignment. Unlike previous phylogenetic placement-based tools such as pplacer and EPA [16, 17], HmmUFOtu makes the assumption that the global topology and branches of the bacterial phylogenetic tree should not be affected by observing an instance of a read, with the exception of adding a novel local branch as necessary for maintaining the correct bi-sectional tree topology (Fig. 2). In essence, the SEP procedure identifies the candidate optimal phylogenetic placement in three steps: first, it finds a candidate list of seed nodes with sequences (observed or inferred) that meet a similarity threshold to the read, as measured by observed p-distance (Fig. 2b, “seed” step). The algorithm then estimates the branching-point, the length of the new branch, and the sub-tree likelihood using the Procedure-Estimate-Place described below (Fig. 2c, “estimate” step). This can be proven to produce near-optimal tree likelihoods and can be performed efficiently since it neither modifies the original reference tree, nor creates an actual copy of the sub-tree (Procedure-Estimate-Place). Finally, estimated placements are sorted by their likelihoods; for every top candidate, a detailed placement algorithm copies the sub-tree, inserts the observed read node according to the aforementioned estimations (Fig. 2e, f), and iteratively optimizes the estimations by maximizing the placement likelihood, or the posterior probability if a prior is used. The final log-likelihood of this subtree is equivalent to the log-likelihood of the entire tree, according to the Pulley Principle [41], as long as a time-reversible DNA substitution model is used (e.g. GTR, TN93, or HKY85 model). This step is further outlined in the Procedure-Joint-Opt-Place described below (Fig. 2g, “place” step).

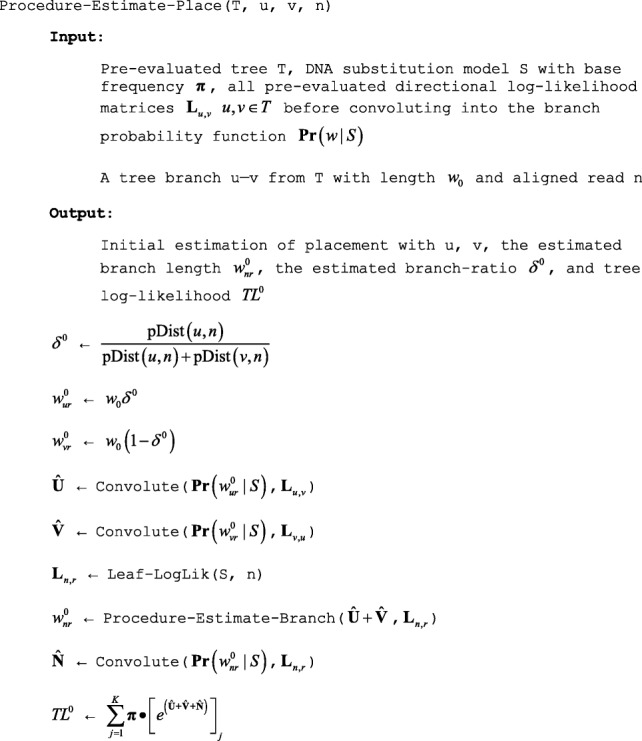



where ● represents the vector’s dot product, **pDist** is the p-distance between the observed or inferred sequence of a node and a read, and **Leaf-LogLik** for evaluating a leaf node as described previously [41]. The **Convolute** is the core, numerical-underflow-free function that calculates the convolution between a node’s log-likelihood matrix whose columns are the conditional log-likelihood vectors, and a branch’s transition probability matrix, and the Procedure-Estimate-Branch is a p-distance-based branch length estimation method, both of which are described in the Appendix.

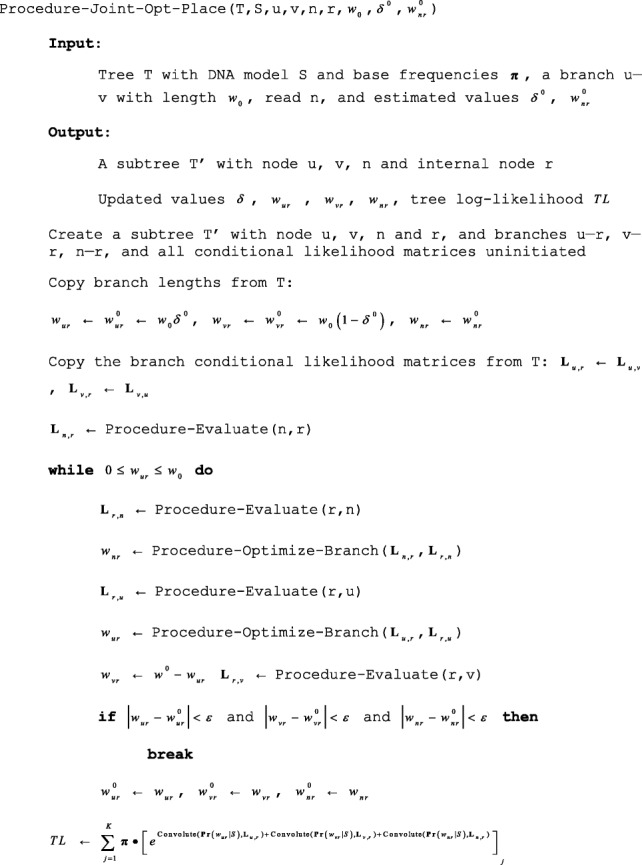



where **Procedure-Evaluate** is the Felsenstein’s pruning algorithm for tree evaluation as mentioned above [41] and **Procedure-Optimize-Branch** for branch-optimization is an EM based algorithm as described previously [41].

### Segment placement comparison algorithm for chimeric read detection

HmmUFOtu implements a “segment placement comparison” algorithm based on principles similar to previously described segment alignment-based methods such as ChimeraSlayer and UCHIME [24, 25]. This algorithm: (1) breaks an aligned sequence (from a read or pair) into an even number of equal sized segments; (2) runs the SEP procedure on every segment using the common set of “seeds” for the entire sequence; (3) picks the best placement for both the 5′ and 3′ segment (5′-best and 3′-best); (4) calculates the alternative placements by re-running the “Estimate” and “Place” procedures for the 5′-segment sequence at the 3′-best branch (5′-alt) and vice versa for the 3′-segment (3′-alt); (5) calculates the joined LOD scores as

$$ LOD=\left({loglik}^{5\hbox{'}- best}-{loglik}^{5\hbox{'}- alt}\right)+\left({loglik}^{3\hbox{'}- best}-{loglik}^{3\hbox{'}- alt}\right) $$

and; (6) identifies potential chimeras where the assigned phylogenetic tree nodes of the 5′-best and 3′-best placements fail to match and the LOD score exceeds a defined cut-off.

### Building HmmUFOtu databases

All HmmUFOtu databases used in our benchmarks were built by the “hmmufotu-build” program using the GreenGenes reference alignment, tree, and taxonomy annotation files. The build process generally consists of building the multiple-alignment index and CSFM-index, training the banded HMM profile, building and evaluating the phylogenetic tree, and optionally estimating the shape parameter of the *dΓ* model for among-site rate variation and subsequent re-evaluation of the tree (Additional file 1: Table S6). The database building process supports multi-threading, with time complexity proportional to the total number of nodes and consensus sites in the reference phylogenetic tree; this process requires ~ 10 mins for building the “gg_97_otus_GTR” database with six processors on the Linux workstation described below.

### Mock bacteria community processing

The mock community bacteria samples were purchased from the America Type Culture Collection (ATCC®) (Catalog # HM-782D). Both the V4 and V1 V3 samples were sequenced as control samples across multiple Illumina MiSeq runs with 150-bp or 300-bp paired-end platforms, respectively. All mock community runs were sequenced by the PENN FGS/NGSC center.

### Estimating model parameters in HmmUFOtu database construction

In order to train an HMM-profile for a given MSA, we adopted the common practice of using Dirichlet-multinomial models as prior distributions of observed transition and emission data [42] as it balances prior biological knowledge with the observed training data. We trained 16S rRNA gene-specific Dirichlet-multinomial models using the GreenGenes gg_97_otus reference dataset for use in our analyses and benchmarking. Specifically, we used Dirichlet-density models (DDs) for transitions and insertion emissions (Fig. 1b, “I” states) and Dirichlet-mixture models (DMs) for the match emissions (Fig. 1b, “M” states). During training, the weights of the observed MSA are normalized using a position-specific method as described previously [42]. Pre-trained Dirichlet-multinomial models are distributed with HmmUFOtu (Additional file 1: Table S7), but users can also train customized models on their own data.

To train the DNA substitution models, we estimated all potential “ancestral-offspring” mutations using the Gojobori 3-sequence method [43] and then estimated the DNA model parameters for the GTR, TN93, and HKY85 models [26, 28, 29]. HmmUFOtu also supports the Goldman two-sequence method [44] for DNA model training as well as many other popular DNA substitution models. The complete list can be found in Additional file 1: Table S8. Pre-trained DNA substitution models are distributed along with HmmUFOtu source code, but users may also train customized DNA models on their own data.

To estimate the shape parameter α of each *dΓ* model used for among-site rate variation in the HmmUFOtu databases we constructed, the 16S rRNA phylogenetic tree was evaluated with one pass using a fixed rate model, then the number of observed mutations occurring at each site is calculated using the known tree topology. The shape parameters were then estimated using the moment matching method, which assumes the parameters follow a negative-binomial distribution, as previously described [32]. The amount of time required for training customized models is negligible compared to building the database. Details of model training can be found in Additional file 1: Table S6.

### Generating phylogeny-based OTUs, consensus-based representative sequences, and OTU-trees

During the OTU summarization step of HmmUFOtu (“hmmufotu-sum”), any phylogenetic tree node containing more than a preset threshold of placed reads across all samples is defined as an OTU containing the NGS reads placed closest to it. To infer the consensus sequences, the posterior probability of observing the four nucleotides at each site is calculated from the observed base counts from aligned reads and a Dirichlet density prior with parameters proportional to the pre-evaluated node likelihood and a concentration parameter of 2. A concentration parameter of N is equivalent to using N unobserved, background “pseudo” reads for each OTU, but this prior can be disabled if set to 0. The consensus base at every site is then inferred by either choosing the base that maximizes the posterior probability or inserting a gap if more gaps were observed. Finally, a phylogenetic tree containing the OTUs is generated by pruning the original reference tree, leaving only nodes defined as an OTU and nodes required to maintain the bi-sectional property of the tree. Specific details of this process can be found in Additional file 1: Table S6.

### Benchmarking settings and data analysis

All benchmarking tests were performed on a Linux workstation with 8 Intel Xeon® processors @ 3.70 GHz and 64 GB RAM, except for the multi-threading performance tests, which were performed on a Linux cluster node with 24 Intel Xeon® processors (12 physical cores) @ 3.07 GHz and 96 GB RAM. Speed and resource statistics are based on the “user” time from system log files. All tested third-party programs were run with the default options, unless specified otherwise. The default methods in the QIIME workflow were: “UCLUST” for OTU-picking, “first” for rep-seq picking, “UCLUST” for assigning taxonomy, “PyNAST” for aligning OTUs, and “FastTree” for constructing the phylogenetic tree of OTUs [6, 45, 46]. All alignment and taxonomic assignment accuracy results were calculated using in-house Perl scripts. For taxonomic assignment accuracy benchmarks, the alignment outputs from HmmUFOtu were used as inputs for both pplacer and EPA to ensure fair comparisons. The alpha-diversity and beta-diversity analyses of mock communities were calculated with QIIME scripts [10] using the observed species and Bray-Curtis metrics, respectively. Details can be found in Additional file 1: Table S6.

### Data requirements and program output

All core programs of HmmUFOtu accept sequences in FASTA or FASTQ format, MSA profiles in FASTA aligned format, and phylogenetic trees in Newick format. The model training programs output in customized plain-text formats (“hmmufotu-train-dm” for Dirichlet prior models, “hmmufotu-train-sm” for DNA substitution models) or in standard HMMER3 text format [18] (“hmmufotu-train-hmm” for HMM profiles). The database building program “hmmufotu-build” outputs databases as custom binary files. The main program “hmmufotu” outputs taxonomy assignments in tab-delimited plain-text format (TSV format). The OTU summarization program “hmmufotu-sum” outputs results in TSV format, which is compatible with third-party tools such as QIIME; it also optionally outputs the rep-seqs in FASTA format and the OTU-tree in Newick format. Optionally, “hmmufotu-jplace” may convert outputted TSV assignment files into “jplace” format for compatibility with third-party tools [47] as long as the dependency C++ library JsonCpp is installed or specified during the configuration step.

### Implementation, dependency, and portability

HmmUFOtu is written in C++ 98 and is only dependent on the header-only libraries of C++ boost and Eigen3 for Newick tree parsing, statistics, and linear-algebra operations. Both libraries are available on most operating systems (OS) and do not require installation. Multi-threading of HmmUFOtu is implemented when native OpenMP is available. All HmmUFOtu tools are built by GNU Autotools allowing for easy installation on most OS including Linux, Mac OS X, and Windows. Pre-compiled HmmUFOtu executables are also available at https://github.com/Grice-Lab/HmmUFOtu/releases.

### System requirements

HmmUFOtu pre-evaluates and stores all the directional conditional log-likelihoods of a reference phylogenetic tree, thus requiring a relatively large amount of memory (RAM) and disk space. For example, the “gg_97_otus_GTR” reference database takes about 20 GB RAM and disk space for both database building and read processing.

### Additional file

Additional file 1: Including supplementary Tables S1–S8 described in this study. (DOCX 74 kb)

## Appendix

where *MIN_EXP* is a scaling constant to avoid numeric-underflow errors.

## Abbreviations

BWT: Burrows-Wheeler transform
CSFM-index: Consensus-sequence Full-Text index
DD: Dirichlet density
DM: Dirichlet mixture
*dΓ*: Discrete-Gamma model
HMM: Hidden Markov model
OS: Operating system
OTU: Operational taxonomic unit
SEP: Seed-Estimate-Place
