# Supplementary material for: HmmUFOtu: An HMM and phylogenetic placement based ultra-fast taxonomic assignment and OTU picking tool for microbiome amplicon sequencing studies
Source: Genome Biol. 2018 Jun 27;19:82. doi: 10.1186/s13059-018-1450-0 (PMC6020470; doi:10.1186/s13059-018-1450-0)
Supplement: Supplementary file 1 — Including supplementary Tables S1–S8 described in this study. (DOCX 74 kb) [file 13059_2018_1450_MOESM1_ESM.docx]

Supplementary Materials

# Supplemental Tables

## Table S1

| Set | Name | Type | Sequence | Strand | CS start | CS end | # Node hits | # Leaf hits | % Note efficiency | % Leaf efficiency |
| --- | --- | --- | --- | --- | --- | --- | --- | --- | --- | --- |
| V4 | 515F | forward | GTGCC  AGCMG  CCGCG  GTAA | + | 465 | 483 | 194,878 | 96,609 | 98.10% | 97.27% |
| V4 | 806R | reverse | GGACT  ACHVG  GGTWT  CTAAT | - | 738 | 757 | 195,208 | 96,591 | 98.27% | 97.25% |
| V1V3 | 27F | forward | AGAGT  TTGAT  CCTGG  CTCAG | + | 14 | 26 | 128,766 | 48,782 | 64.82% | 49.12% |
| V1V3 | 534R | reverse | ATTAC  CGCGG  CTGCT  GG | - | 468 | 484 | 191,634 | 94,938 | 96.47% | 95.59% |
| V3V5 | 357F | forward | CCTAC  GGGAG  GCAGC  AG | + | 311 | 327 | 184,379 | 91,527 | 92.82% | 92.15% |
| V3V5 | 926R | reverse | CCGTC  AATTC  MTTTR  AGT | - | 862 | 879 | 195,858 | 97,185 | 98.60% | 97.85% |

***Table S1.*** *Theoretical annealing efficiency calculated by HmmUFOtu (hmmufotu-anneal) of three different sets of bacterial 16S rRNA gene sequencing primers, based on the “gg_97_otus_GTR” database. CS start and CS end: start and end positions relative to the 16S rRNA gene consensus sequence (CS); Leaves: observed OTU nodes with known sequences; Nodes: Leaves and internal ancestor tree nodes with phylogenetically inferred sequences. Only nodes with >=90% sequence similarity (p-Distance) to both forward and reverse primers are considered as “hits”.*

## Table S2

| ID | Taxonomy | #  species | Relative abundance |
| --- | --- | --- | --- |
| NC_009085 | k__Bacteria;p__Proteobacteria;c__Gammaproteobacteria;o__Pseudomonadales;f__Moraxellaceae;g__Acinetobacter | 1 | 0.05 |
| NZ_AAYI02000000 | k__Bacteria;p__Actinobacteria;c__Actinobacteria;o__Actinomycetales;f__Actinomycetaceae;g__Actinomyces | 1 | 0.05 |
| NC_003909 | k__Bacteria;p__Firmicutes;c__Bacilli;o__Bacillales;f__Bacillaceae;g__Bacillus | 1 | 0.05 |
| NC_009614 | k__Bacteria;p__Bacteroidetes;c__Bacteroidia;o__Bacteroidales;f__Bacteroidaceae;g__Bacteroides | 1 | 0.05 |
| NC_009617 | k__Bacteria;p__Firmicutes;c__Clostridia;o__Clostridiales;f__Clostridiaceae;g__Clostridium | 1 | 0.05 |
| NC_001263 | k__Bacteria;p__Thermi;c__Deinococci;o__Deinococcales;f__Deinococcaceae;g__Deinococcus | 1 | 0.05 |
| NC_17316 | k__Bacteria;p__Firmicutes;c__Bacilli;o__Lactobacillales;f__Enterococcaceae;g__Enterococcus | 1 | 0.05 |
| NC_000913 | k__Bacteria;p__Proteobacteria;c__Gammaproteobacteria;o__Enterobacteriales;f__Enterobacteriaceae | 1 | 0.05 |
| NC_000915 | k__Bacteria;p__Proteobacteria;c__Epsilonproteobacteria;o__Campylobacterales;f__Helicobacteraceae;g__Helicobacter | 1 | 0.05 |
| NC_008530 | k__Bacteria;p__Firmicutes;c__Bacilli;o__Lactobacillales;f__Lactobacillaceae;g__Lactobacillus | 1 | 0.05 |
| NC_003210 | k__Bacteria;p__Firmicutes;c__Bacilli;o__Bacillales;f__Listeriaceae | 1 | 0.05 |
| NC_003112 | k__Bacteria;p__Proteobacteria;c__Betaproteobacteria;o__Neisseriales;f__Neisseriaceae;g__Neisseria | 1 | 0.05 |
| NC_006085 | k__Bacteria;p__Actinobacteria;c__Actinobacteria;o__Actinomycetales;f__Propionibacteriaceae;g__Propionibacterium | 1 | 0.05 |
| NC_002516 | k__Bacteria;p__Proteobacteria;c__Gammaproteobacteria;o__Pseudomonadales;f__Pseudomonadaceae;g__Pseudomonas | 1 | 0.05 |
| NC_007493 | k__Bacteria;p__Proteobacteria;c__Alphaproteobacteria;o__Rhodobacterales;f__Rhodobacteraceae;g__Rhodobacter | 1 | 0.05 |
| NC_010079 | k__Bacteria;p__Firmicutes;c__Bacilli;o__Bacillales;f__Staphylococcaceae;g__Staphylococcus | 2 | 0.1 |
| NC_004116 | k__Bacteria;p__Firmicutes;c__Bacilli;o__Lactobacillales;f__Streptococcaceae;g__Streptococcus | 3 | 0.15 |

***Table S2.*** *Mock community bacterial taxonomy information and theoretical composition. All the mock community information is based on the product information from American Type Culture Collection (ATCC) catalog number HM-782.*

## Table S3

| Sample name | Subject ID | Visit number | Body site | Study name | File name | Data type |
| --- | --- | --- | --- | --- | --- | --- |
| LAH_1 | 159591683 | 1 | left auriculotemporal skin | WGS-PP1 | SRS013258.scaffolds.fa.bz2 | wgs_assembled |
| LAH_1 | 159591683 | 1 | left auriculotemporal skin | WGS-PP1 | SRS013258.fsa.gz | trimmed_16s |
| LAH_2 | 159591683 | 2 | left auriculotemporal skin | WGS-PP1 | SRS024596.scaffolds.fa.bz2 | wgs_assembled |
| LAH_2 | 159591683 | 2 | left auriculotemporal skin | WGS-PP1 | SRS024596.fsa.gz | trimmed_16s |
| RAH_1 | 159591683 | 1 | right auriculotemporal skin | WGS-PP1 | SRS013261.scaffolds.fa.bz2 | wgs_assembled |
| RAH_1 | 159591683 | 1 | right auriculotemporal skin | WGS-PP1 | SRS013261.fsa.gz | trimmed_16s |
| RAH_2 | 159591683 | 2 | right auriculotemporal skin | WGS-PP1 | SRS024598.scaffolds.fa.bz2 | wgs_assembled |
| RAH_2 | 159591683 | 2 | right auriculotemporal skin | WGS-PP1 | SRS024598.fsa.gz | trimmed_16s |
| Gingva_1 | 159268001 | 1 | gingiva | WGS-PP1 | SRS013723.scaffolds.fa.bz2 | wgs_assembled |
| Gingva_1 | 159268001 | 1 | gingiva | WGS-PP1 | SRS013723.fsa.gz | trimmed_16s |
| Gingva_2 | 159268001 | 2 | gingiva | WGS-PP1 | SRS023938.scaffolds.fa.bz2 | wgs_assembled |
| Gingva_2 | 159268001 | 2 | gingiva | WGS-PP1 | SRS023938.fsa.gz | trimmed_16s |
| ExternalNaris_1 | 159490532 | 1 | external naris | WGS-PP1 | SRS017044.scaffolds.fa.bz2 | wgs_assembled |
| ExternalNaris_1 | 159490532 | 1 | external naris | WGS-PP1 | SRS017044.fsa.gz | trimmed_16s |
| ExternalNaris_2 | 159490532 | 2 | external naris | WGS-PP1 | SRS044474.scaffolds.fa.bz2 | wgs_assembled |
| ExternalNaris_2 | 159490532 | 2 | external naris | WGS-PP1 | SRS044474.fsa.gz | trimmed_16s |
| BuccalMucosa_1 | 159814214 | 1 | buccal mucosa | WGS-PP1 | SRS017215.scaffolds.fa.bz2 | wgs_assembled |
| BuccalMucosa_1 | 159814214 | 1 | buccal mucosa | WGS-PP1 | SRS017215.fsa.gz | trimmed_16s |
| BuccalMucosa_2 | 159814214 | 2 | buccal mucosa | WGS-PP1 | SRS043422.scaffolds.fa.bz2 | wgs_assembled |
| BuccalMucosa_2 | 159814214 | 2 | buccal mucosa | WGS-PP1 | SRS043422.fsa.gz | trimmed_16s |

***Table S3.*** *Summary of the Human Microbiome Project (HMP) dataset used for real data benchmarking by HmmUFOtu. All HMP datasets were downloaded from the HMP FTP deposit (*[*ftp://public-ftp.ihmpdcc.org/*](ftp://public-ftp.ihmpdcc.org/)*).*

## Table S4

| pDist range | T | P | TPR | TNR | PPV | ACC |
| --- | --- | --- | --- | --- | --- | --- |
| [0.01,0.03) | 10000 | 5024 | 91.94% | 79.50% | 81.91% | 85.75% |
| [0.03,0.05) | 10000 | 4876 | 94.54% | 82.96% | 84.08% | 88.61% |
| [0.05,0.1) | 10000 | 4970 | 95.65% | 86.74% | 87.70% | 91.17% |
| [0.1,0.15) | 10000 | 5055 | 94.80% | 89.28% | 90.04% | 92.07% |

***Table S4.*** *Chimera read detection performance using simulated dataset “gg_97_otus_chimera”. Chimera cross-over points are restricted in [0.25, 0.75] of the consensus sequences of 16S rRNA reference sequences from the GreenGene 97% OTU references. pDist: p-distance between the two reference sequences forming the chimeras. T, P: total and positive (chimera) reads in this pDist range, respectively; TPR: sensitivity, TNR: specificity, PPV: precision, ACC: accuracy. All values based on the minimum LOD cut-off of zero (0) for chimera detection.*

## Table S5

| Dataset | | | Performance measurement | | | | | | | | | | | | | | Speed | |
| --- | --- | --- | --- | --- | --- | --- | --- | --- | --- | --- | --- | --- | --- | --- | --- | --- | --- | --- |
|  |  |  | **kingdom** | | **phylum** | | **class** | | **order** | | **Family** | | **genus** | | **species** | |  |  |
| GTR | TPR | 100.00% | | 99.96% | | 99.51% | | 99.25% | | 99.02% | | 95.48% | | 82.40% | | 1.96 | |  |
|  | TNR | nan | | 94.63% | | 89.18% | | 94.87% | | 97.67% | | 97.82% | | 99.27% | |  |  |  |
|  | PPV | 100.00% | | 99.98% | | 99.51% | | 99.26% | | 98.91% | | 95.36% | | 82.56% | |  |  |  |
|  | ACC | 99.98% | | 99.92% | | 99.03% | | 98.68% | | 98.57% | | 97.05% | | 98.58% | |  |  |  |
| TN93 | TPR | 100.00% | | 99.96% | | 99.49% | | 99.25% | | 99.02% | | 95.37% | | 82.47% | | 1.96 | |  |
|  | TNR | nan | | 95.08% | | 90.16% | | 95.26% | | 97.81% | | 97.94% | | 99.30% | |  |  |  |
|  | PPV | 100.00% | | 99.98% | | 99.55% | | 99.32% | | 98.98% | | 95.61% | | 83.16% | |  |  |  |
|  | ACC | 99.98% | | 99.92% | | 99.07% | | 98.73% | | 98.62% | | 97.10% | | 98.61% | |  |  |  |
| HKY85 | TPR | 100.00% | | 99.96% | | 99.51% | | 99.29% | | 99.07% | | 95.40% | | 82.70% | | 1.93 | |  |
|  | TNR | nan | | 94.85% | | 89.80% | | 95.11% | | 97.78% | | 97.85% | | 99.27% | |  |  |  |
|  | PPV | 100.00% | | 99.98% | | 99.53% | | 99.30% | | 98.96% | | 95.44% | | 82.51% | |  |  |  |
|  | ACC | 99.98% | | 99.92% | | 99.07% | | 98.74% | | 98.64% | | 97.05% | | 98.59% | |  |  |  |
| GTR + dΓ  (0.491) | TPR | 100.00% | | 99.94% | | 99.36% | | 99.06% | | 97.76% | | 90.76% | | 71.14% | | 1.23 | |  |
|  | TNR | nan | | 95.75% | | 81.86% | | 92.50% | | 97.28% | | 97.68% | | 99.21% | |  |  |  |
|  | PPV | 100.00% | | 99.98% | | 99.17% | | 98.92% | | 98.71% | | 94.85% | | 79.07% | |  |  |  |
|  | ACC | 99.98% | | 99.90% | | 98.58% | | 98.21% | | 97.58% | | 95.45% | | 98.07% | |  |  |  |
| TN93 + dΓ  (0.492) | TPR | 100.00% | | 99.96% | | 99.37% | | 99.03% | | 97.92% | | 91.62% | | 72.16% | | 1.21 | |  |
|  | TNR | nan | | 95.30% | | 84.69% | | 93.60% | | 97.61% | | 97.57% | | 99.22% | |  |  |  |
|  | PPV | 100.00% | | 99.98% | | 99.30% | | 99.08% | | 98.87% | | 94.66% | | 79.40% | |  |  |  |
|  | ACC | 99.98% | | 99.92% | | 98.71% | | 98.32% | | 97.80% | | 95.65% | | 98.12% | |  |  |  |
| HKY85 + dΓ  (0.492) | TPR | 100.00% | | 99.96% | | 99.41% | | 99.11% | | 97.97% | | 91.51% | | 71.19% | | 1.19 | |  |
|  | TNR | nan | | 95.08% | | 83.67% | | 93.26% | | 97.61% | | 97.61% | | 99.23% | |  |  |  |
|  | PPV | 100.00% | | 99.98% | | 99.25% | | 99.03% | | 98.87% | | 94.74% | | 79.46% | |  |  |  |
|  | ACC | 99.98% | | 99.92% | | 98.70% | | 98.35% | | 97.83% | | 95.64% | | 98.09% | |  |  |  |

***Table S5.*** *Taxonomy assignment performance and speed comparison between different DNA substitution models and fixed or dΓ among-site rate variation models benchmarked with the V4 simulated dataset. TPR: sensitivity, TNR: specificity, PPV: precision, ACC: accuracy. “Nan” indicates no observed data. Speed is measured as reads per second per processor. GTR model: Generalized time-reversible model; TN93: Tamura and Nei 1993 model; HKY85: Hasegawa, Kishino and Yano 1985 model; dΓ: discrete Gamma model of among-site rate variation. Values in parentheses show the estimated shape parameters of the dΓ models.*

## Table S6

| Program type | Program name | Options | Explanation |
| --- | --- | --- | --- |
| Database building | hmmufotu-build |  | build an HmmUFOtu database |
|  |  | 97_otus.fasta | MSA alignment input from GreenGenes |
|  |  | 97_otus.tree | tree input from GreenGenes |
|  |  | -n gg_97_otus | use this database name |
|  |  | -a 97_otus_taxonomy.txt | taxonomy annotation input from GreenGenes |
|  |  | -p 6 | number of threads/processors |
| Model training | hmmufotu-train-dm |  | train a Dirichlet prior model |
|  |  | 97_otus.fasta | MSA alignment input from GreenGenes |
|  |  | -o gg_97_otus.dm | Dirichlet model output |
|  |  | -n 5 | number of different random seed to try in training |
|  | hmmufotu-train-sm |  | train a DNA substitution model |
|  |  | 97_otus.fasta | MSA alignment input |
|  |  | 97_otus.tree | tree input |
|  |  | -s GTR | DNA model type |
|  |  | -m Gojobori | model training method |
| Read simulating | hmmufotu-sim |  | Generating simulated reads from a database |
|  |  | gg_97_otus_GTR | database name |
|  |  | -N 5000 | number of desired reads |
| Taxonomic assignment | hmmufotu |  | performing per-read taxonomic assignment |
|  |  | gg_97_otus_GTR | database name |
|  |  | R1.fastq (R2.fastq) | FASTQ read input file(s) |
|  |  | -o hmmufotu_assignment.txt | taxonomic assignment output |
|  |  | -p 6 | number of threads/processors |
|  |  | -a hmmufotu_alignment.fasta | write the sequence alignments in additional FASTA output |
| Chimera read detection | hmmufotu | --chimera | enable chimera detection |
|  |  | --chimera-lod | min LOD cut-off |
|  |  | --chimera-info | include detailed chimera information in output |
|  |  | --chimera-out hmmufotu_filtered.txt | keep filtered chimera read assignments in separate output |
| OTU summarization | hmmufotu-sum |  | generate an OTU table from taxonomic assignment results |
|  |  | gg_97_otus_GTR | database name |
|  |  | hmmufotu_assignment.txt | assignment input(s) |
|  |  | -o hmmufotu_OTU.txt | OTU table output |
|  |  | -c hmmufotu_OTU_CS.fasta | consensus sequence output for each OTU |
|  |  | -t hmmufotu_OTU.tree | Newick tree output for OTUs |
|  |  | -r hmmufotu_OTU_id.txt | OTU-read ID map output |
| Inspecting and exporting hmmufotu database | hmmufotu-inspect |  | inspect and optionally export database data for third-party tools |
|  |  | gg_79_otus_GTR | database name |
|  |  | -s gg_79_otus_GTR.fasta | export the reference sequences with internal IDs and taxonomy info |
|  |  | -t gg_79_otus_GTR.tree | export the reference tree with internal IDs |
| Third-party tool phylogenetic placement | pplacer |  | pplacer described in [1] |
|  |  | hmmufotu_alignment.fasta | aligned sequence input generated by hmmufotu |
|  |  | -t gg_79_otus_GTR.tree | reference tree input with HmmUFOtu internal IDs |
|  |  | -r gg_79_otus_GTR.fasta | aligned reference sequences with HmmUFOtu internal IDs |
|  |  | -j 6 | number of threads/processors |
|  |  | -o pplacer_placement.jplace | phylogenetic placement output in jplace format |
|  | raxmlHPC-PTHREADS-SSE3 |  | RAxML tool with the EPA algorithm included [2] |
|  |  | -f v | running the EPA phylogenetic placement algorithm |
|  |  | -m GTRCAT | using GTR model and substitution rate optimization |
|  |  | -V | disable rate heterogeneity among site |
|  |  | -t gg_79_otus_GTR.tree | reference tree input with HmmUFOtu internal IDs |
|  |  | -n EPA_placement | job name for outputs |
|  |  | -T 6 | number of threads/processors |
| Community analysis | biom convert |  | QIIME script for converting OTU table format [3] |
|  |  | -i hmmufotu_OTU.txt | OTU table input from hmmufotu-sum |
|  |  | -o hmmufotu_OTU.biom | OTU table output in BIOM format |
|  |  | --table-type=”OTU table” | input format |
|  |  | --to-hdf5 | output format |
|  | alpha_diversity.py |  | QIIME script for calculating community alpha-diversity [3] |
|  |  | -i hmmufotu_OTU.biom | OTU table input |
|  |  | -o hmmufotu_OTU_alpha.txt | alpha-diversity output |
|  |  | -m observed_species | metric for alpha-diversity |
|  | beta_diversity.py |  | QIIME script for calculating community beta-diversity [3] |
|  |  | -i hmmufotu_OTU_ref.biom | OTU table input casted into reference taxa |
|  |  | -o hmmufotu_OTU_beta | beta-diversity output dir |
|  |  | -m bray_curtis, weighted_unifrac | metrics for beta-diversity |

***Table S6.*** *Programs and running options used in this study. Source or Linux versions of executables were used for all programs on a Linux Workstation or Linux cluster. All input and output file names have been shortened for clarity.*

## Table S7

|  | | Dirichlet mixture category | | | | |
| --- | --- | --- | --- | --- | --- | --- |
|  |  | 1 | 2 | 3 | 4 | 5 |
| Concentration parameter | A | 1.055 | 0.822 | 6.501 | 279.2 | 0.8358 |
|  | C | 53.61 | 0.848 | 3.243 | 2.659 | 2.013 |
|  | G | 1.348 | 1.0118 | 156.5 | 4.274 | 0.8160 |
|  | T | 2.8157 | 0.7920 | 2.824 | 2.689 | 38.25 |
| Mixture coefficient | | 0.1558 | 0.3302 | 0.2292 | 0.1651 | 0.1197 |

***Table S7.*** *Pre-trained Dirichlet-multinomial prior model for 16S rRNA genes in GreenGene 97% OTU reference using HmmUFOtu. The model is trained using an EM algorithm from “hmmufotu-train” by trying 5 different random seeds. Category 3 and 4 stand for strongly G-rich and A-rich regions existing in certain 16S rRNA genes in the training set, respectively; Category 2 represents the degenerative sites without base preference.*

## Table S8

|  |  | *Parameters in HmmUFOtu* | *# of free parameters* | *Explanations* |
| --- | --- | --- | --- | --- |
| *DNA substitution models* | *GTR* | *α,β,γ,δ,ε,η,A,C,G,T* | *8* | *Generalized time-reversible model [4]* |
|  | *TN93* | *β,κ_r_,κ_y_,A,C,G,T* | *5* | *Differentiate between bases, 2 transitions and transversion [5]* |
|  | *HKY85* | *β, κ,A,C,G,T* | *4* | *Differentiate between bases, transition and transversion [6]* |
|  | *F81* | *β,A,C,G,T* | *3* | *Differentiate between bases [7]* |
|  | *K80* | *β, κ* | *1* | *Differentiate between transition and transversion [8]* |
|  | *JC69* |  | *0* | *All equal rates* |
| *Rate variation* | *dΓ* | *α,π,K* | *5K-1* | *K is the number of Dirichlet mixture categories* |

***Table S8.*** *Models for DNA substitution and among-site variation supported by HmmUFOtu. All parameters are related to mutation rate matrix (Q) and base frequencies (π). Free parameters are determined by the total base frequency restriction (A+C+G+T=1) and by measuring time in substitutions.*

# Supplemental References

1. Matsen FA, Kodner RB, Armbrust EV: **pplacer: linear time maximum-likelihood and Bayesian phylogenetic placement of sequences onto a fixed reference tree.** *BMC Bioinformatics* 2010, **11:**538.

2. Berger SA, Krompass D, Stamatakis A: **Performance, accuracy, and Web server for evolutionary placement of short sequence reads under maximum likelihood.** *Syst Biol* 2011, **60:**291-302.

3. Caporaso JG, Kuczynski J, Stombaugh J, Bittinger K, Bushman FD, Costello EK, Fierer N, Pena AG, Goodrich JK, Gordon JI, et al: **QIIME allows analysis of high-throughput community sequencing data.** *Nat Methods* 2010, **7:**335-336.

4. Waddell PJ, Steel MA: **General time-reversible distances with unequal rates across sites: mixing gamma and inverse Gaussian distributions with invariant sites.** *Mol Phylogenet Evol* 1997, **8:**398-414.

5. Tamura K, Nei M: **Estimation of the number of nucleotide substitutions in the control region of mitochondrial DNA in humans and chimpanzees.** *Mol Biol Evol* 1993, **10:**512-526.

6. Hasegawa M, Kishino H, Yano T: **Dating of the human-ape splitting by a molecular clock of mitochondrial DNA.** *J Mol Evol* 1985, **22:**160-174.

7. Felsenstein J: **Evolutionary trees from DNA sequences: a maximum likelihood approach.** *J Mol Evol* 1981, **17:**368-376.

8. Kimura M: **A simple method for estimating evolutionary rates of base substitutions through comparative studies of nucleotide sequences.** *J Mol Evol* 1980, **16:**111-120.
